# Study protocol of the Going to Stay at Home program: evaluation of a residential carer training program to reduce dementia carer distress and burden

**DOI:** 10.1186/2193-1801-3-330

**Published:** 2014-07-01

**Authors:** Meredith Gresham, Ruby S M Tsang, Megan Heffernan, Henry Brodaty

**Affiliations:** HammondCare, New South Wales, Australia; Dementia Collaborative Research Centre, School of Psychiatry, Faculty of Medicine, University of New South Wales, Sydney, Australia; Centre for Healthy Brain Ageing, School of Psychiatry, Faculty of Medicine, University of New South Wales, Sydney, Australia; Academic Department of Old Age Psychiatry, Prince of Wales Hospital, Avoca Street, Randwick, NSW Australia; Dementia Collaborative Research Centre, University of New South Wales, AGSM Building, Sydney, NSW 2052 Australia

**Keywords:** Dementia, Carers, Caregivers, Training, Psychosocial intervention, Residential respite

## Abstract

**Background:**

Caring for a person with dementia has profound physical, psychological, social and financial impacts on the carer, while morbidity in carers has detrimental effects on outcomes in people with dementia. A 10-day hospital-based residential carer training program (BMJ 299(6712):1375–1379, 1989) delayed residential care placement, delayed mortality, reduced carer’s psychological morbidity and lowered care costs. This study aims to evaluate the effects of a similar program adapted for use with residential respite.

**Methods/Design:**

This is a single-arm longitudinal study conducted at a residential aged care facility involving 100 people with dementia and their primary carers. In a 7-day residential program, carers will attend intensive training sessions while the people with dementia are engaged in daily activities. Data will be collected at the start of the residential program (baseline), at 6 months (post 1) and at 12 months (post 2) after completion of the program. The primary outcome is carer psychological distress. Secondary outcomes include carer burden, carer quality of life and time to residential care placement.

**Discussion:**

This study will provide evidence on the effectiveness of the program in reducing carer distress and burden as well as delaying institutionalisation of the person with dementia, which may have important implications for policy.

## Background

Approximately 70% of people with dementia in Australia live in the community and most of them receive some informal care (AIHW 2012). Informal carers play a pivotal role in the lives of people with dementia living in the community. They provide assistance with core and non-core activities, either continuously or for more than 40 hours per week (Ory et al. 1999; AIHW 2012). The majority of people with dementia have a ‘profound’ level of disability and this places a high demand on the carer (AIHW 2012). Consequently, dementia carers often experience greater burden than carers of people with other chronic conditions or disabilities (Ory et al. 1999), and the experience of providing care has been described as an ‘unremitting burden’ (Anderson 1987).

Carer burden refers to the carer’s perception of the extent to which their physical, psychological, social and financial status has been affected as a result of care provision (Zarit et al. 1986). High levels of burden have been consistently reported in carers of people with dementia (Schulz et al. 1995; Baumgarten et al. 1992; Haley et al. 1987; Pinquart and Sörensen 2003) and such burden may persist up to two years after the person with dementia’s admission to formal care (Waltrowicz et al. 1996). Longitudinally, depression severity increased in carers who received usual care only while it decreased in those who received additional counselling and support (Mittelman et al. 2008). Mean level of burden and percentage of carers reporting a high level of burden also increased across time (Brodaty et al. 2013). Besides effects on psychological well-being, dementia caregiving also has significant adverse effects on the carer’s physical health (Vitaliano et al. 2003). Carers of people with dementia generally report poorer health (Haley et al. 1987; Baumgarten et al. 1992) and greater use of health services (Haley et al. 1987; Kiecolt-Glaser et al. 1991) than their non-caregiving counterparts.

The level of burden experienced by carers can be influenced by a myriad of factors. Behavioural and psychological symptoms of dementia (BPSD) (e.g., aggression, agitation) have been shown to be associated with carer burden (Black and Almeida 2004; Donaldson et al. 1998). Moreover, carers who reported lower satisfaction with the social support they receive or had greater reactions to the person with dementia’s behavioural problems had more symptoms of depression (Mittelman et al. 2008). The association between severity of the illness (either cognitive or functional impairment) and carer burden, however, was weak (Brodaty and Hadzi-Pavlovic 1990). Carer outcomes may also be influenced by the specific coping strategies used or the level of self-efficacy (Saad et al. 1995; Kneebone and Martin 2003; Fortinsky et al. 2002; Gilliam and Steffen 2006).

Adverse carer outcomes can lead to adverse outcomes in people with dementia. For instance, high levels of carer burden is a consistent significant predictor of institutionalisation (Yaffe et al. 2002; Gaugler et al. 2003; Hébert et al. 2001), and carer psychological morbidity was also a significant predictor of mortality of the care recipient (Brodaty et al. 1993). Therefore, carer interventions may benefit both the carer and the person with dementia. Carer interventions have significant but modest effects in improving carer knowledge, reducing carer burden, reducing psychological morbidity and delaying institutionalisation (Brodaty et al. 2003; Pinquart and Sörensen 2006). There is evidence that these effects can be maintained for up to 11 months post-intervention (Pinquart and Sörensen 2006). In particular, a 10-day Prince Henry Hospital residential carer training program proved effective in delaying residential care placement, delaying mortality, reducing carer’s psychological morbidity and lowering care costs (Brodaty and Gresham 1989; Brodaty and Peters 1991; Brodaty et al. 1993, 1997). These results suggest that carer training provided early in the course of dementia has significant benefits on the outcomes of people with dementia, and therefore such intensive carer training programs should be made more extensively available.

Residential respite care is defined as alternative, short-term care in a residential aged care facility (RACF) with the main purpose of providing the family carer with respite and support. The use of residential respite care has steadily increased in Australia, but take up rates remain low, with only 32% of eligible people with dementia accessing residential respite care services within 12 months of gaining approval (AIHW 2012). There appears to be little empirical support for the efficacy of residential respite care, with some studies even reporting adverse effects on people with dementia, such as an increase in problem behaviours and earlier institutional placement (Brodaty and Gresham 1992). However, anecdotal reports from carers suggest residential respite care may have significant benefits provided carers are well informed and the most appropriate services are utilised. Residential respite care can provide an opportunity to combine existing knowledge about carer interventions with respite.

The principal aim of this study is to evaluate, in an open single-arm trial, the effects of the *Going to Stay at Home* program, which is adapted from the Prince Henry Hospital program for use with residential respite.

It is hypothesised that:

1. carer psychological distress will be reduced at 6- and 12-month follow-up compared to baseline,
2. carer burden will be reduced at 6- and 12-month follow-up compared to baseline,
3. there will be no increase in BPSD in the person with dementia, and
4. a minimum of 60% of carers will have the person with dementia still living at home at 12-month follow-up.

## Methods/Design

### Study design

A single-arm longitudinal design will be used. Data will be collected at three time points: at the start of the residential program (baseline), at 6 months (post 1) and at 12 months (post 2) after completion of the program.

### Setting

The study will be conducted in an eight-bedroom cottage at an RACF in southern Sydney, Australia.

### Recruitment of participants

People with dementia and their primary carers enrolled in the *Going to Stay at Home* program at the RACF will be invited to participate in this evaluation study. Participants of the GTSAH program will be recruited through the community care division of the RACF, aged care assessment teams, carer groups and hospitals, and will then be clustered into groups of four to six person with dementia-carer dyads according to their demographic characteristics.

Inclusion criteria for people with dementia:

- confirmed diagnosis of dementia
- living at home with a primary carer
- able to understand and communicate in English
- informed consent (self and/or proxy) and verbal assent to participate

Exclusion criterion for people with dementia:

- no written consent and/or verbal assent to participate

Inclusion criteria for carers:

- able to understand and communicate in English
- informed consent, and where appropriate, consent for the person with dementia to participate

Exclusion criterion for carers:

- no written consent to participate

### Intervention

The 7-day residential program will begin on a Sunday and finish on a Saturday (see Table 1).

**Table 1:** **Timetable of the program for carers**

	Morning	Afternoon	Evening
Sunday		• Welcome and orientation	Socialising
Monday	• Combating isolation	• Medical aspects of dementia	Socialising
		• Relaxation	
Tuesday	• Fitness	• Communication	Socialising
	• Reminiscence and reality orientation	• Assertion	
Wednesday	• Re-roling	• Therapeutic use of activities	Socialising
	• Nutrition	• Work simplification, organisation and safety at home	
Thursday	• Nursing skills	• Planning for the future	Carers’ outing
		• Using community services	
Friday	• Caring for self	• *Carers’ choice of topic*	Socialising
	• Relaxation		
Saturday	• Farewell		

#### Program for carers

There will be 14 intensive psychoeducation sessions for carers. These sessions will be delivered in an informal structure, with didactic segments, group work, modelling and role play. The sessions cover the following topics:

1. *Combating isolation*. Carers will be encouraged to share their experiences of the caring role to establish an open, supportive relationship among carers.
2. *Medical aspects of dementia*. This session will provide information on the causes of different types of dementia, behavioural and psychiatric changes that may occur, and other common comorbidities including depression and delirium.
3. *Planning for the future*. The need to plan for future emergencies and unforseen events (e.g., unplanned hospitalisation or death of the carer) will be discussed in this session. Other issues such as driving, legal and financial matters, as well as Advance Care Planning will also be explored.
4. *Re-roling*. This session will explore the issues pertaining to the changes in roles and responsibilities between the person with dementia and the carer. Practical problem solving skills will be taught to help carers encourage the person with dementia to allow the carer to take over some of the tasks while maintaining his/her dignity and to develop the carer’s confidence in taking over these roles.
5. *Reminiscence and reality orientation*. Carers will be provided with information on techniques of reminiscence (e.g. use of a life history book), environmental reality orientation (e.g. use of signs or clocks) and validation to enable the person with dementia to maintain connection with their environment as well as improve the communication between the carer and the person with dementia.
6. *Communication*. Information on receptive and expressive dysphasia that the person with dementia may experience and how these may affect communication will be provided, and then carers will have the opportunity to learn communication strategies to enhance understanding.
7. *Assertion*. Carers will be provided with an understanding of assertive, non-assertive and aggressive behaviours, a “carer’s bill of rights” and strategies to cope with criticisms to ensure that they are heard and understood and their personal rights are maintained.
8. *Therapeutic use of activities*. Activities that may be meaningful and enjoyable to the person with dementia will be identified in this session and carers will be provided with knowledge of activity analysis that enables them to break down familiar tasks into component steps and to modify or eliminate steps that prevent the person with dementia from completing them.
9. *Work simplification, organisation and safety in the home*. Problem solving skills of prioritising, simplifying and using outside assistance will be explored to help carers achieve a balance of work, leisure and rest in their daily life. Tools to identify risks within the home environment will be provided and safety measures to minimise the risk to the person with dementia will be discussed.
10. *Nursing skills*. Clinical issues associated with dementia such as incontinence, medication, mobility, personal care and acute illness will be covered and carers will receive hands-on experience in using clinical care equipment that can be used to aid care practices.
11. *Fitness*. Information about the benefits of exercise for both the person with dementia and the carer will be provided, and a daily exercise routine will be established.
12. *Nutrition*. Changes in diet, food intake and nutritional needs of the person with dementia will be examined, and carers will be provided with practical skills to enhance intake as well as improve the eating experience.
13. *Caring for self*. This session will provide carers with the opportunity to identify their need for support and they will be taught a variety of relaxation and stress management techniques.
14. *Using community services*. Information about the types of support services available in the community as well as how to contact and access these services will be provided.

#### Program for people with dementia

The program for people with dementia will include activities that focus on sensory stimulation, environmental orientation, brain training, physical activity, reminiscence, self-expression, creativity, social interactions and relaxation. Outings may be arranged depending on personal preferences. A large variety of activities will be arranged to cater to different personal preferences and individual needs. The people with dementia will also be provided with the opportunity to discuss changes in their memory and their brain on a physiological level depending on their ability and willingness to receive the information.

### Assessment

Global cognitive function of people with dementia will be assessed at baseline by RACF staff using the Mini Mental State Examination (MMSE) (Folstein et al. 1975).

Outcome and additional measures will be collected at all three time points: baseline, post 1 and post 2. Information will be collected through self-report and proxy questionnaires, as well as through interviews with the carer.

#### Outcome measures

The primary outcome is carer psychological distress measured using the 10-item Kessler Psychological Distress Scale (K-10) (Kessler et al. 2002). The K-10 is a self-report measure of global psychological distress based on questions about anxiety and depressive symptoms; higher scores indicate greater distress. The version used in this study employs a referent time frame of ‘the last four weeks’ instead of ‘the last 30 days’ in the original questionnaire (ABS 2003).

Secondary outcomes are carer burden, carer quality of life, time to residential care placement, carer functional health status, carer needs, resource utilisation, person with dementia instrumental activities of daily living, person with dementia activities of daily living, person with dementia quality of life, person with dementia neuropsychiatric symptoms, person with dementia agitation and dementia severity.

- *Carer burden*: The 12-item Zarit Burden Interview, short version (ZBI-12) (Bédard et al. 2001; Zarit et al. 1980) is a self-report measure of burden experienced in the caring role. The ZBI-12 has been shown to yield results comparable to those of the original full version (Bédard et al. 2001). Higher scores indicate greater burden.
- *Carer quality of life*: The 13-item Older People’s Quality of Life Questionnaire – Brief (OPQoL-Brief) (Bowling et al. 2013) will be used to measure quality of life. Higher scores indicate better quality of life.
- *Time to residential care placement*: Time between recruitment and residential care placement of the person with dementia will be measured in days.
- *Carer functional health status*: The 12-item Short Form Health Survey, version 2 (SF-12v2) (Ware et al. 1996) will be used to assess carers’ functional health status in the physical and mental domains; higher scores indicate better health.
- *Carer needs*: A 11-item self-report questionnaire (Harrison et al. 2013) covering various areas on which carers for people with dementia may need information will be used to assess the met and unmet needs of the carer.
- *Resource utilisation*: The Resource Utilization in Dementia – Lite instrument (RUD Lite) (Wimo and Winblad 2003) will be used to assess the utilisation of both formal and informal care and the associated costs. This is administered as an informant-report questionnaire rather than through an informant interview.
- *Instrumental activities of daily living (IADL)*: The Instrumental Activities of Daily Living Scale (IADL) (Lawton and Brody 1969) will be used to assess people with dementia’s competence in performing eight types of IADL. This will be administered in the form of an informant-report questionnaire.
- *Activities of daily living (ADL)*: The Physical Self-Maintenance Scale (PSMS) (Lawton and Brody 1969) will be used to assess people with dementia’s competence in six types of behaviours including toileting, feeding, dressing, grooming, physical ambulation and bathing. The version used here separates toileting into two items covering bladder and bowel control. This will be administered as an informant-report questionnaire.
- *Quality of life*: The proxy version of the 13-item Quality of Life – AD (QoL-AD) (Logsdon et al. 1999, 2002) will be used to assess the quality of life of people with dementia.
- *Neuropsychiatric symptoms*: The 12-item Neuropsychiatric Inventory (NPI) (Cummings et al. 1994) is an informant-rated measure of the severity and frequency of behavioural and psychiatric disturbances in the people with dementia, collected through an interview with the carer. Higher scores indicate more BPSD.
- *Agitation*: The 37-item Cohen-Mansfield Agitation Inventory – Community (CMAI-C) (Cohen-Mansfield et al. 1995) is an informant-rated measure of the frequency of agitated behaviours in the people with dementia, collected through an interview with the carer. Higher scores indicate greater agitation.
- *Dementia severity*: The Global Deterioration Scale (GDS) (Reisberg et al. 1982) will be used to classify people with dementia based on the relative severity of their cognitive impairment and their function in activities of daily living; higher scores indicate more severe dementia. This will be rated by the staff of the respite facility.

### Interview and focus group

Carers will be asked to describe the effects of the program and which aspects they found most helpful. Focus groups will be held with managerial staff to discuss the impact of the program on facility-wide practices and the potential for similar programs to continue within the facility.

### Statistical analysis

#### Sample size

A target sample size of 100 person with dementia-carer dyads has been estimated to provide over 80% power to detect a moderate effect size (Cohen’s d = 0.3) difference at a significance level of 0.05 (two-sided), assuming a medium within-subject correlation (ρ = 0.5) between pre- and post-intervention measures.

#### Outcome analyses

The difference in outcome measures before and after intervention will be examined using a paired *t*-test at a significance level of 0.05 (two-sided). To control for covariates (NPI severity, dementia severity) linear mixed models will be used.

#### Missing data

Patterns of missing data will be examined to identify potential biases. Where applicable, missing items will be handled as suggested in the literature for that particular instrument.

### Ethics

This study has been approved by the Human Research Ethics Committee, University of New South Wales, Sydney, Australia (Approval No.: #HC13033). Written consent will be obtained from all participating people with dementia and their primary carers. In the case where the person with dementia is deemed unable to provide informed consent, a responsible person will provide written consent and verbal assent will be obtained from the person with dementia.

## Discussion

*Going to Stay at Home* is a 7-day residential carer training program, which was adapted for use in residential aged care settings from a successful hospital-based residential carer training program. The present study aims to evaluate the effects of this program. Outcomes for carers and people with dementia will be measured using standardised instruments. In addition, qualitative analyses of interview and focus group data will be carried out to determine which components of the program carers found most effective and the impact of the program on practices within the residential aged care facility. This evaluation study will provide evidence for whether the program is effective in reducing carer psychological distress and burden as well as delaying institutionalisation of the person with dementia, which may have important implications for policy.

## References

[CR1] (2003). Information paper: use of the Kessler psychological distress scale in ABS health surveys, Australia, 2007–08.

[CR2] (2012). Dementia in Australia.

[CR3] Anderson R (1987). The unremitting burden on carers. BMJ.

[CR4] Baumgarten M, Battista RN, Infante-Rivard C, Hanley JA, Becker R, Gauthier S (1992). The psychological and physical health of family members caring for an elderly person with dementia. J Clin Epidemiol.

[CR5] Bédard M, Molloy DW, Squire L, Dubois S, Lever JA, O’Donnell M (2001). The Zarit burden interview: a new short version and screening version. Gerontologist.

[CR6] Black W, Almeida OP (2004). A systematic review of the association between the behavioral and psychological symptoms of dementia and burden of care. Int Psychogeriatr.

[CR7] Bowling A, Hankins M, Windle G, Bilotta C, Grant R (2013). A short measure of quality of life in older age: the performance of the brief older people’s quality of life questionnaire (OPQOL-brief). Arch Gerontol Geriatr.

[CR8] Brodaty H, Gresham M (1989). Effect of a training programme to reduce stress in carers of patients with dementia. BMJ.

[CR9] Brodaty H, Gresham M (1992). Prescribing residential respite care for dementia—effects, side-effects, indications and dosage. Int J Geriatr Psychiatry.

[CR10] Brodaty H, Hadzi-Pavlovic D (1990). Psychosocial effects on carers of living with persons with dementia. Aust N Z J Psychiatry.

[CR11] Brodaty H, Peters KE (1991). Cost effectiveness of a training program for dementia carers. Int Psychogeriatr.

[CR12] Brodaty H, McGilchrist C, Harris L, Peters KE (1993). Time until institutionalization and death in patients with dementia: role of caregiver training and risk factors. Arch Neurol.

[CR13] Brodaty H, Gresham M, Luscombe G (1997). The Prince Henry Hospital dementia caregivers’ training programme. Int J Geriatr Psychiatry.

[CR14] Brodaty H, Green A, Koschera A (2003). Meta-analysis of psychosocial interventions for caregivers of people with dementia. J Am Geriatr Soc.

[CR15] Brodaty H, Woodward M, Boundy K, Ames D, Balshaw R (2013). Prevalence and predictors of burden in caregivers of people with dementia. Am J Geriatr Psychiatry.

[CR16] Cohen-Mansfield J, Werner P, Watson V, Pasis S (1995). Agitation among elderly persons at adult day-care centers: the experiences of relatives and staff members. Int Psychogeriatr.

[CR17] Cummings JL, Mega M, Gray K, Rosenberg-Thompson S, Carusi DA, Gornbein J (1994). The Neuropsychiatric Inventory: comprehensive assessment of psychopathology in dementia. Neurology.

[CR18] Donaldson C, Tarrier N, Burns A (1998). Determinants of carer stress in Alzheimer’s disease. Int J Geriatr Psychiatry.

[CR19] Folstein MF, Folstein SE, McHugh PR (1975). “Mini-mental state”: a practical method for grading the cognitive state of patients for the clinician. J Psychiatr Res.

[CR20] Fortinsky RH, Kercher K, Burant CJ (2002). Measurement and correlates of family caregiver self-efficacy for managing dementia. Aging Ment Health.

[CR21] Gaugler JE, Kane RL, Kane RA, Clay T, Newcomer R (2003). Caregiving and institutionalization of cognitively impaired older people: utilizing dynamic predictors of change. Gerontologist.

[CR22] Gilliam CM, Steffen AM (2006). The relationship between caregiving self-efficacy and depressive symptoms in dementia family caregivers. Aging Ment Health.

[CR23] Haley WE, Levine EG, Brown SL, Berry JW, Hughes GH (1987). Psychological, social, and health consequences of caring for a relative with senile dementia. J Am Geriatr Soc.

[CR24] Harrison F, Low L-F, Barnett A, Gresham M, Brodaty H (2013). What do clients expect of community care and what are their needs? The community care for the elderly: needs and service use study (CENSUS). Australas J Ageing.

[CR25] Hébert R, Dubois M-F, Wolfson C, Chambers L, Cohen C (2001). Factors associated with long-term institutionalization of older people with dementia: data from the Canadian Study of Health and Aging. J Gerontol A Biol Sci Med Sci.

[CR26] Kessler RC, Andrews G, Colpe LJ, Hiripi E, Mroczek DK, Normand S-LT, Walters EE, Zaslavsky AM (2002). Short screening scales to monitor population prevalences and trends in non-specific psychological distress. Psychol Med.

[CR27] Kiecolt-Glaser JK, Dura JR, Speicher CE, Trask OJ, Glaser R (1991). Spousal caregivers of dementia victims: longitudinal changes in immunity and health. Psychosom Med.

[CR28] Kneebone II, Martin PR (2003). Coping and caregivers of people with dementia. Br J Health Psychol.

[CR29] Lawton MP, Brody EM (1969). Assessment of older people: self-maintaining and instrumental activities of daily living. Nurs Res.

[CR30] Logsdon RG, Gibbons LE, McCurry SM, Teri L (1999). Quality of life in Alzheimer’s disease: patient and caregiver reports. J Ment Health Aging.

[CR31] Logsdon RG, Gibbons LE, McCurry SM, Teri L (2002). Assessing quality of life in older adults with cognitive impairment. Psychosom Med.

[CR32] Mittelman MS, Brodaty H, Wallen AS, Burns A (2008). A three-country randomized controlled trial of a psychosocial intervention for caregivers combined with pharmacological treatment for patients with Alzheimer disease: effects on caregiver depression. Am J Geriatr Psychiatry.

[CR33] Ory MG, Hoffman RR, Yee JL, Tennstedt S, Schulz R (1999). Prevalence and impact of caregiving: a detailed comparison between dementia and nondementia caregivers. Gerontologist.

[CR34] Pinquart M, Sörensen S (2003). Differences between caregivers and noncaregivers in psychological health and physical health: a meta-analysis. Psychol Aging.

[CR35] Pinquart M, Sörensen S (2006). Helping caregivers of persons with dementia: which interventions work and how large are their effects?. Int Psychogeriatr.

[CR36] Reisberg B, Ferris SH, de Leon MJ, Crook T (1982). The global deterioration scale for assessment of primary degenerative dementia. Am J Geriatr Psychiatry.

[CR37] Saad K, Hartman J, Ballard C, Kurian M, Graham C, Wilcock G (1995). Coping by the carers of dementia sufferers. Age Ageing.

[CR38] Schulz R, O’Brien AT, Bookwala J, Fleissner K (1995). Psychiatric and physical morbidity effects of dementia caregiving: prevalence, correlates, and causes. Gerontologist.

[CR39] Vitaliano PP, Zhang J, Scanlan JM (2003). Is caregiving hazardous to one’s physical health? A meta-analysis. Psychol Bull.

[CR40] Waltrowicz W, Ames D, McKenzie S, Flicker L (1996). Burden and stress on relatives (informal carers) of dementia sufferers in psychogeriatric nursing homes. Aust J Ageing.

[CR41] Ware JE, Kosinski M, Keller SD (1996). A 12-item short-form health survey: construction of scales and preliminary tests of reliability and validity. Med Care.

[CR42] Wimo A, Winblad B (2003). Resource utilization in dementia: RUD Lite. Brain Aging.

[CR43] Yaffe K, Fox P, Newcomer R, Sands L, Lindquist K, Dane K, Covinsky KE (2002). Patient and caregiver characteristics and nursing home placement in patients with dementia. JAMA.

[CR44] Zarit SH, Reever KE, Bach-Peterson J (1980). Relatives of the impaired elderly: correlates of feelings of burden. Gerontologist.

[CR45] Zarit SH, Todd PA, Zarit JM (1986). Subjective burden of husbands and wives as caregivers: a longitudinal study. Gerontologist.

